# Supplementary material for: A Food Relief Charter for South Australia—Towards a Shared Vision for Pathways Out of Food Insecurity
Source: Int J Environ Res Public Health. 2022 Jun 9;19(12):7080. doi: 10.3390/ijerph19127080 (PMC9222515; doi:10.3390/ijerph19127080)
Supplement: Supplementary file 1 [file ijerph-19-07080-s001.zip › ijerph-1735881-supplementary.pdf]

# South Australian Food Relief Charter

## Preamble

Food is essential for life and access to sufficient, safe, culturally appropriate, nutritious food is a basic human right.

Food security is a key determinant of health, with long term food insecurity linked to chronic diseases such as diabetes and heart disease.

Individuals and families may experience periods of food insecurity for many reasons, and whilst these may stem from crisis, for many, food insecurity becomes a chronic issue.

Food can be a non-threatening mechanism to engage people in opportunities and services that build skills and capacity to support them to move out of food insecurity.

## Commitment of Partners

The South Australian Food Relief Charter has been co-designed in partnership between the food relief sector and government. It represents a commitment to working collaboratively towards an optimal food relief system that leads to improved client and community outcomes.

The signatories of this Charter are committed to working in partnership to:

i. increase food and nutrition security for South Australians and improve health outcomes

ii. build sector wide standards for best practice in the food relief system

iii. create opportunities for people to build skills and capacity to move out of food insecurity

iv. build a skilled and sustainable food relief sector workforce

We recognise that food and nutrition security is achieved by having access to a safe, affordable, culturally appropriate and nutritious food supply at all times.

## Guiding Principles

As signatories to this Charter, the following principles will guide our future actions and endeavours to provide best practice service delivery to people experiencing food insecurity.

### **Principle 1**: Collaborating to build an effective and integrated food relief system

Maximise the collective impact of the food relief sector by co-designing enhanced models of practice that continue to deliver an invaluable service to South Australians in need, through recognising and addressing the underlying causes of food insecurity.

**Why this is important:**

The food relief sector plays a crucial role in empowering people to move from poverty and exclusion to connection and participation in life. However, the food relief system is comprised of many different agencies, with different roles, approaches and geographic coverage. Improving coordination of advocacy and services across the system increases the likelihood of reducing the number of people reliant on food relief over the long term.

## **Principle 2**: Focusing on nutrition and health

Maximise the availability of healthy and appropriate food through the implementation of the *'Nutrition Guidelines for the Food Relief Sector in South Australia'* and support individuals and families to improve their food and nutrition by fostering opportunities to increase skills and knowledge.

**Why this is important:**

Food insecurity is linked to chronic diseases in adulthood, including obesity, diabetes, and heart disease. Prolonged food insecurity can result in poor nutrition and other factors that affect children's normal growth and development, including educational outcomes. The provision of food relief provides a valuable opportunity to improve health outcomes among vulnerable populations by maximising the availability of high quality, healthy foods and minimising the provision of unhealthy food and drinks.

## **Principle 3**: Delivering a service built on fairness and equity

Ensure safe, affordable, culturally appropriate and nutritious food is available to everyone in need. Service delivery includes values such as choice, safety, dignity, respect, compassion, transparency, privacy, cultural sensitivity, empowerment and independence.

**Why this is important:**

People experiencing food insecurity generally access food relief services as a last resort and often experience shame and embarrassment regarding the need to access these services. A respectful, values-based service culture upholds client dignity, the provision of choice and supports clients to move out of food insecurity.

## **Principle 4**: Connecting people, building skills and confidence

Food can be a non-threatening mechanism for engagement. Engagement through food can open the way for connection to other services and support social connections that help address the complex interrelated issues which have contributed to food insecurity. This can assist in facilitating a pathway out of food insecurity.

**Why this is important:**

For many people, food insecurity becomes a chronic problem, not simply an acute crisis resolved with a food parcel. Low income, unemployment, a reliance on social assistance, housing affordability, chronic ill health, and high living costs are all determinants of both poverty and food insecurity. While food relief is critical to supporting people in crisis, other wrap-around services are crucial to reducing long-term reliance and providing a pathway out of food insecurity.

## **Principle 5**: Monitoring and evaluating to measure collective impact

Enhance the collective impact and value of the sector through the development of a shared outcomes framework, which ensures that relevant information and data is collected and shared to quantify and qualify outputs and outcomes on an ongoing basis.

**Why this is important:**

Collecting and sharing information and data assists with delivering better, more effective services that meet the needs of the clients, and addresses and minimises overlap and gaps in service delivery. It supports continuous improvement for the sector and can identify opportunities for agencies to draw and build on each other's experience.

Government of South Australia
